# Collagen Fibrillogenesis in the Mitral Valve: It’s a Matter of Compliance

**DOI:** 10.3390/jcdd8080098

**Published:** 2021-08-20

**Authors:** Richard L. Goodwin, Arash Kheradvar, Russell A. Norris, Robert L. Price, Jay D. Potts

**Affiliations:** 1 Department of Biomedical Sciences, School of Medicine Greenville, University of South Carolina, Greenville, SC 29605, USA; 2 Department of Biomedical Engineering, The Henry Samueli School of Engineering, University of California, Irvine, CA 92697, USA; arashkh@uci.edu; 3 Department of Regenerative Medicine, School of Medicine, Medical University of South Carolina, Charleston, SC 29425, USA; norrisra@musc.edu; 4 Department of Cell Biology and Anatomy, School of Medicine, University of South Carolina, Greenville, SC 29605, USA; bob.price@uscmed.sc.edu (R.L.P.); jay.potts@uscmed.sc.edu (J.D.P.)

**Keywords:** mitral valve, collagens, fibrillogenesis

## Abstract

Collagen fibers are essential structural components of mitral valve leaflets, their tension apparatus (chordae tendineae), and the associated papillary muscles. Excess or lack of collagen fibers in the extracellular matrix (ECM) in any of these structures can adversely affect mitral valve function. The organization of collagen fibers provides a sophisticated framework that allows for unidirectional blood flow during the precise opening and closing of this vital heart valve. Although numerous ECM molecules are essential for the differentiation, growth, and homeostasis of the mitral valve (e.g., elastic fibers, glycoproteins, and glycans), collagen fibers are key to mitral valve integrity. Besides the inert structural components of the tissues, collagen fibers are dynamic structures that drive outside-to-inside cell signaling, which informs valvular interstitial cells (VICs) present within the tissue environment. Diversity of collagen family members and the closely related collagen-like triple helix-containing proteins found in the mitral valve, will be discussed in addition to how defects in these proteins may lead to valve disease.

## 1. Collagen in the Mitral Valve

The mechanical integrity of the mitral valve largely depends on its ECM composition, as the cells provide only little to no mechanical strength [1]. Along with elastic fibers, collagen fibers are the principal protein components of the valve ECM. While the elastic fibers play an essential role in mitral valve function, it is indeed the collagen fibers with the proper tensile strength to maintain tissue integrity during peak systole (~120 mmHg blood pressure applied over the surface of the human mitral valve). Additional structural components of the valve ECM include hydrophilic glycoprotein and glycan molecules that occupy turgid water-filled spaces in valve tissue. These hydrophilic molecules can occupy large regions of the valve leaflet as they do in humans or relatively little as is the case in the mouse mitral valve. They also work in concert with the collagen fibers in valve tissue throughout the cardiac cycle, particularly in chordae tendineae where they are thought to be essential for the molecular crimping (waving) and uncrimping (straightening) of these fibers as they are loaded and unloaded during systole and diastole [2].

**Figure 1:**
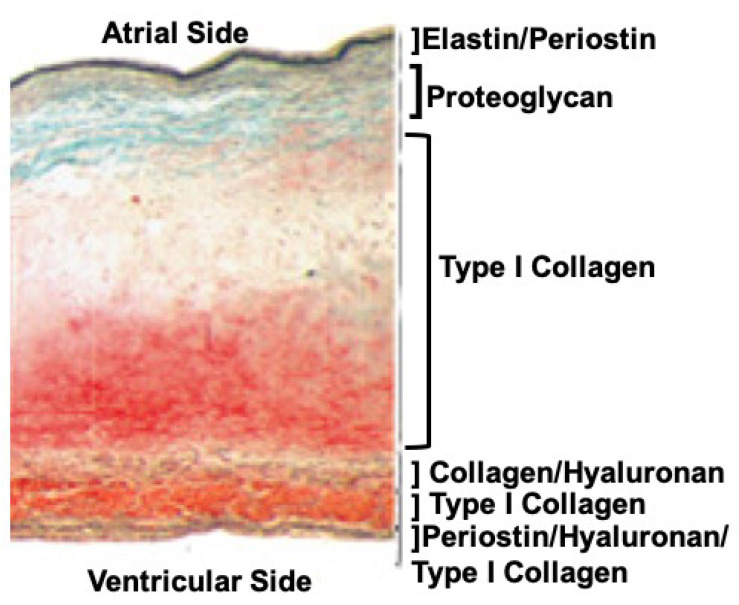
Cross-section of a normal human mitral valve leaflet stained with Movats reveals a sophisticated tissue architecture that can be categorized into at least six layers based on the ECM composition alone. Although collagen fibers are found throughout these layers, their structures (primary, secondary, and tertiary), and the proteins they associate with, can vary. Image was modified from [3].

Traditionally, valve leaflet tissue architecture is described as a three-layered structure, where the flow side contains elastic fibers and the non-flow side includes collagen fibers that sandwich a water laden middle layer, the spongiosa, acting in a cushion-like fashion to absorb the compressive forces due to coaptation. While conceptually useful, the model is not sufficient to fully appreciate the diversity that exists in different valve leaflets of the diverse animal model systems. For instance, the mouse mitral valve in some regions, is only 3–5 cells thick, with no clear demarcation between them, whereas the human mitral valve’s leaflets are hundreds of cells thick and can be categorized into six distinct zones according to their ECM composition (Figure 1) [3].

While it is macroscopically unclear why the mammalian mitral valve has evolved into a unique bileaflet valve, mitral valve and its components are microscopically far more sophisticated than a simple, three-layered structure. For instance, collagen fibers are ubiquitous throughout all six layers of the valve leaflets, chordae, and the supporting papillary muscles. However, the density, diameter and subunits of the collagen fibers appear to be specific for each tissue subtype within the mitral valve [4]. It appears that collagen fibers’ diameter, orientation, and composition are adjusted for specific functions of papillary muscle tissue, chordae or the valve leaflets [2]. Further, we posit that this fine tuning of fibrillogenesis is carried out by valvular interstitial cells (VICs), which have genetic regulatory systems that rely on mechanical feedback to build and maintain the specific collagen architecture for each leaflet. VICs are fibroblast-like cells of mesenchymal origin that produce and remodel the mitral valve tissues’ ECM including all six layers of the leaflets and the chordae throughout the lifespan. Therefore, disruptions in either the genetic regulatory mechanisms or changes in mechanical forces can lead to adverse or maladaptive collagen fiber remodeling and ultimately mitral dysfunction. 

## 2. Collagens, Fibrils, and Fibers of the Mitral Valve

“Collagen” is derived from the Greek word “kolla”, which means glue. Collagen proteins, as their name implies, are the glue that holds multicellular animals together. In fact, the diversification and expansion of the collagen gene family from the ancestral type IV-like collagen gene have been proposed to have enabled animal multicellularity and tissue evolution [5]. Since type IV collagen is normally found in the basement membranes’ ECM, it seems that the evolution of this structure was pivotal in the evolution of multicellularity. We suggest that the evolution of the collagen fibril-forming collagens provided the tensile strength necessary for the high-pressure perfusion systems found in tetrapods. In addition to those found in cardiac tissues, collagen fibrils are also found throughout the vascular system, and in prominent larger vessels, normally confined to the outer adventitial layer.

**Figure 2:**
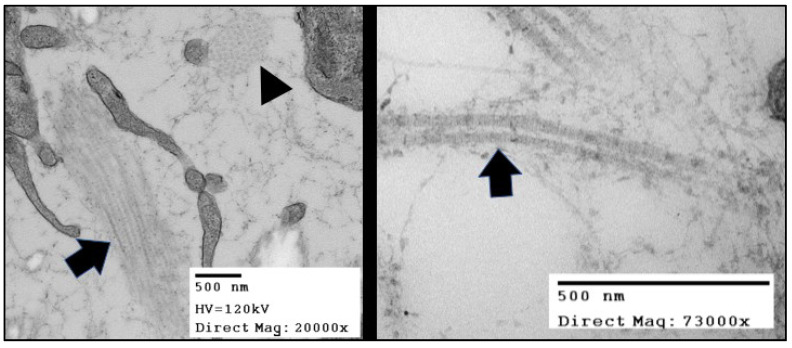
TEMs of the collagen fibers in in Hamburger Hamilton (HH) stage 42 chick (*Gallus gallus*) valve leaflets. The image in the right panel shows bundles of collagen fibrils arranged in two distinct originations. The fiber oriented in the plane of the figure (black arrow) displays the “D-period” striations. The fiber oriented into the plane of the figure (black arrowhead) shows the quasi-hexagonal arrangement of collagen fibers in cross-section.

Collagen fibrils are collections of fibril-forming collagen molecules made of three alpha chains that form an intertwined triple helix. Collagen molecules auto assemble into hetero- or homotypic trimers of collagen propeptides while still in the endoplasmic reticulum. The C-terminal propeptides drive the nucleation of the triple helix as the trimer zips together from the C- to the N-terminus [6]. The procollagen molecules undergo additional processing around the time of deposition into the ECM environment. The removal of the procollagen domains by specific N- and C-terminal proteinases triggers fibrillogenesis, or the spontaneous polymerization of collagen molecules into fibrils. The C-terminal proteases are critical regulators of fibril formation, whereas the N-terminal proteinases are thought to regulate fibril shape and diameter [7]. As fibrils are formed, these macromolecular complexes can be recognized with transmission electron microscopy and display the classic D-periodic (65–67 nm) banding pattern (Figure 2). These fibrils then form higher order structures by bundling together into collagen fibers that vary in length and diameter depending on the tissue.

In humans, the 28 collagen types are encoded by 43 genes with only types I, II, III, V, and XI form fibrils. All of these fibril-forming collagens are expressed in mitral valves during some point in development. Since fibrils can be composed of mixtures of these collagen types, it is likely that different combinations of collagen types confer different mechanical properties to the ECM of tissues. In the mitral valve, collagen fibrils in the atrial-facing elastic layer have combinations of types I, III, and V that are distinct from fibrils of the ventricular side of the leaflet and chordae [8].

The so-called fibril-associated collagens with interrupted triple helices, or FACITs, do not form fibrils but function to stabilize and connect fibrils to the other ECM components and resident cells. Of these types, IX, XII, and XIV have been found to be expressed in the mitral valve. Other collagen proteins such as type VI collagen directly interact with fibrils and regulate fibrillogenesis. In a process that resembles that of its namesake tendons, the large diameter, highly aligned, collagen fibers of the chordae develop into regular dense connective tissue in association with Col VI. Col VI is an unusual member of the collagen family that forms distinctive beaded microfilaments in the ECM. It appears to have a critical role in preserving the biomechanical properties of tissues that are under high physiological demand such as tendons and the mitral chordae [9]. 

During the development of mitral valve, cushions expand and elongate into functional leaflets. The chordae sculpted off the myocardial wall and remain largely composed of cardiomyocytes with very little ECM between them [3]. As the valve tissues mature, the cells of the chordae begin to express increasing amounts of periostin and fibril-forming collagens such as type I [10]. Interestingly, periostin expression is most intense at the myocardial tendinous junction. In mice, only 2 to 3 chordae are present at birth, whereas adults have 10 to 15 chordae. 

Anatomically, in adults, the chordae emerge out of the anteriomedial and posteriomedial papillary muscles of the left ventricle (LV) with a few sparse collagen fibers found within myocardium of the papillary muscles. Large collagen fibers isotropically arrange within the inner core of the chordae to provide the major tensile strength. The collagen fibers of the inner core are wrapped by two layers of elastin [11], creating a unique viscoelastic tissue with complex mechanical properties. The anterior and posterior mitral leaflets have marginal, basal, and struts of the chordae that connect the leaflets to the papillary muscles. These chordae vary in diameter, with the marginal chordae having the smallest and the strut chordae having the largest diameter. These chordae also differ in collagen composition and organization, with marginal chordae having a smaller fibril diameter and a higher fibril density, while strut chordae exhibit a larger average fibril diameter and a lower fibril density [2]. Accordingly, the different chordae types vary in their mechanical behaviors, with the marginal chordae being less extensible and stiffer than basal and strut chordae [12]. This indicates that the individual chordae may have specific roles during mitral valve coaptation.

A feature of the collagen fibers of the chordae is their ‘wavy’ or ‘crimped’ appearance when imaged while unloaded. This crimped appearance of collagen fibers is seen in other regular dense connective tissues such as in the tendons of the skeletomuscular system, and, as mentioned above, is associated with type VI collagen [9]. In the mitral valve of larger vertebrates, crimped collagen fibers are found in all the chordae and throughout the collagen dense regions of the leaflets, though in different periodicities [2].

**Figure 3:**
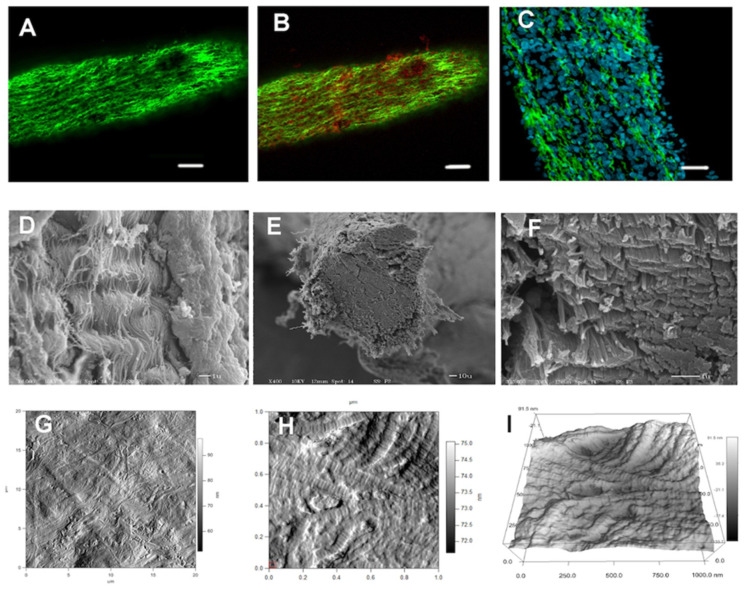
Microscopic examination of chordae tendineae from HH stage 44 chicken embryos (*Gallus gallus*). Various forms of microscopy were used to analyze the structure and composition of murine chordae. The top panel images (**A**–**C**) were obtained using laser scanning confocal microscopy. The collagen distribution in the chordae was determined. (**A**) Represents the chordae stained with an antibody to type I collagen (green). The extensive wavy pattern of the fibrils is observed. (**B**) The same section stained with an antibody to type VI collagen (red). The type VI collagen is observed dispersed near the type I collagen. (**C**) A higher magnification image of the chordae showing the nuclei of the cells (blue) and the extreme wavy collagen type I fibers (green). The middle panel contains images of a similar chordae obtained using scanning electron microscopy (SEM). (**D**) The image shows the surface of the chordae with the underlying collagen fibrils in an organized wavy orientation. (**E**) The chordae were cut in a cross-sectional manner to visualize the alignment of the collage fibers running the length of the chordae. (**F**) Higher magnification showing the cut ends of the collagen fibers and the tightly organized arrangement of the fibers. The lower panel contains images obtained using atomic force microscopy. (**G**) The surface of the chordae was visualized in tapping mode to show the general contour of the surface. (**H**) A higher magnification of the same area showing the individual collagen fibers and the “D-period” periodicity of a single fiber. (**I**) A 3D rendering of the surface topology of the cord using the data generated from the previous image. The scale bars in A–B equal 100 μm, C equal 20 μm. All other images contain scale information.

It appears that this crimped collagen fiber conformation forms relatively late (perinatal) during the heart development and is presaged by type VI collagen expression. Type VI collagen is expressed early in AV valve development and is deposited in an anisotropic pattern. Later, this pattern changes to isotropic and is aligned with the direction of tension in the chordae (Figure 3). It is during this time of valve development that the collagen fibers take on the crimped morphology.

Where the chordae connect to the mitral valve’s anterior and posterior leaflets, collagen fiber orientation becomes more complex. Moving toward the annulus, the mitral leaflets thicken with collagen fibers being found in the atrial-facing elastic layer, and more densely on the ventricular side the leaflets. Collagen fibers are found in all six zones of the mitral leaflet, though they differ in density and organization (Figure 1) [3]. In the atrial-facing elastic zone, collagen fibers form a basket-like shape, with the fibers running circumferentially with sheets of elastin interwoven radially. Periostin is co-expressed with type I collagen. Collagen fibers become sparse in the proteoglycan-rich zone but are abundant in the thick, collagen-rich middle layer of the leaflets. In the next three layers, collagen fibers are found associated with hyaluronic acid, in a hyaluronic acid-free and periostin-free zone and finally, in a zone in which they are associated with periostin and hyaluronic acid. This last zone faces the ventricular side of the mitral leaflets with the largest and densest collagen fibers of the valve leaflets providing the most structural support for the leaflets.

While the mechanisms that regulate collagen fiber orientation are not well understood, there is evidence that collagen fibers are capable of long-range (up to 1 mm) signaling, enabling tension-driven fiber alignment in fibrous matrixes [13]. By constant stretching and relaxation, collagen fibers significantly contribute to the structural integrity of the mitral valve to hold up to the recurrent loads during the cardiac cycles [14]. As a well-known fact, the mechanical response of a heart valve’s leaflet to cyclical loads is regulated by the collagen fiber’s capacity to align with the direction of the principal stresses applied to that leaflet [15,16,17]. 

In the hyaluronic acid and hyaluronic acid-free and periostin-free zones, type II collagen is expressed abundantly during development and throughout the lifespan in mice [18]. The expression of type II collagen is notable as this fiber-forming collagen is almost exclusively associated with cartilaginous tissues. It has been proposed that cardiac valve development shares aspects of cartilage, tendon, and bone development with similar genetic mechanisms [19].

To directly test the role of flow on ECM development in early valve tissues, Tan et al., used a dynamic 3D culturing system [20]. Although it was only in the creeping-flow range (0.86 mm/sec), these cells responded by depositing type I collagen via a Rho/periostin-driven mechanism [20]. Similar experiments using physiological and even supra physiological levels of flow found similar results in outflow valve anlagen, though the mechanisms appear to be slightly different in that Rac signaling appeared to be more predominant in these precursor cells [21]. While this study sheds light on the effect of flow on ECM development, it is not yet known how more sophisticated ventricular flows such as vortices [22] in the left ventricle affect mitral valve cell viability and ECM maintenance.

## 3. Collagen Fibers and Mitral Dysfunction

Creating the optimal collagen fibers’ architecture for the mitral valve function depends on VIC’s ability to produce those and the biomechanical feedback they receive. If either is compromised, valve pathology may ensue. Clearly, deleterious mutations in any of the fibril-forming collagens can lead to mitral valve pathology. This is the case in osteogenesis imperfecta where a mutation in a fibril-forming collagen, Col I, drives the debilitating bone defect as well as mitral valve prolapse (MVP). Mutations in these genes are highly penetrant and lethal. Rupture of the chordae are common in MVP, which implicates the VICs’ inability to maintain the requisite collagen fiber mechanics.

The phenotypes associated with mutations in other genes are initially subtle but can result in severe pathology over time. In Marfan syndrome, a mutation in fibrillin-1 interferes with elastin expression and TGFb signaling. While asymptomatic early in life, a significant number of these patients develop MVP and accompanying myxomatous disease of the valve leaflet and chordae pathology. During this sequela, mitral leaflets elongate and thicken over time. Histologically, the leaflets acquire dense areas of excessive collagen fibers that surround large accumulations of proteoglycans. Matrix fiber fragmentation is also a common feature of this disorder [23]. Murine and porcine models of Marfan also develop MVP with myxomatotic valves [24,25].

Ehlers-Danlos syndrome (EDS) is a collection of connective tissue disorders that has been recently reviewed elsewhere [26]. Some EDS patients also develop myxomatous mitral valves. In a rare disorder, a COL1A2 mutation results in a cardiac valvular form of EDS [27]. Here, as in osteogenesis imperfecta, a mutation in a fibril-forming collagen results in MVP [28].

Prolapse of the mitral valve can result in mitral regurgitation (MR), in which blood flows back into the left atrium during systole. Some of the MR cases are due to either Barlow’s disease or Fibroelastic deficiency (FED) [29]. In Barlow’s disease, the leaflets are myxomatous with the MR involves both leaflets but chordae rupture is rare in these patients [30]. In contrast, in FED, the leaflets are thin, and chordae rupture is more common with the regurgitation involving a limited number of segments of a single leaflet. While Barlow’s disease and FED are clearly clinically discernable, their histopathology is not as straightforward [23,31]. It has been suggested that these two forms of MR are part of a spectrum of a single mitral valve degenerative disease that can focally affect the valve as in FED or more broadly as in Barlow’s disease [31]. In both FED and Barlow’s disease, VICs remodel the ECM of both leaflet and chordae as they are responsible for the pathophysiological adaptation process.

In all the above, VICs are not able to produce the correct ECM structural components. This results in a mismatch of material properties within the valve tissues and cardiac physiology, which leads to rupture of the chordae among other defects. Pathological ECM remodeling occurs as the VICs receive mechanical feedback that the matrix needs to be modified.

In rheumatic heart disease (RHD), untreated streptococcal infections lead to fibrotic mitral leaflets and MVP through mechanisms that are not well defined. Accordingly, the fibrosis is likely due to the chronically inflamed tissues. Presence of inflammatory cells, such as macrophages and neutrophils, releasing a cytokine storm is likely to stimulate VIC to overexpress fibrillar collagens. Unfortunately, RHD continues to be a global problem that is completely preventable [32]. 

Mitral dysfunction may also occur due to defects in the mechanical feedback systems to the VICs. A key cellular structure in VICs that regulates ECM production is primary cilia [33]. These immotile cilia are built around a 9 + 0 microtubular structure that are critical for a number of essential cell-signaling pathways including hedgehog, Wnt, and integrins. Intriguingly, defects in these pathways as well as proteins that regulate primary cilia formation and maintenance can all lead to the MVP phenotype.

## 4. Conclusions

Collagen fibers in the mitral valve are highly heterogeneous. They vary in subunit composition, diameter, density, and morphology. These fiber characteristics appear to be tailored to the specific function of each valve component (e.g., marginal chordae vs. strut chordae) by VICs. It is hypothesized that competent VICs assemble specific collagen fibers in response to the mechanical environment. How VICs receive and respond to this mechanical information is largely unknown, but of critical importance.
